# On the Healing of Abscesses by the First Intention

**Published:** 1855-08

**Authors:** 


On the Healing of Abscesses by the First Intention. By M. Chaissaignac.

M. Chaissaignac has for some time past endeavored to unite abscesses by the first intention, after their complete evacuation; and he reports that his success has been very considerable. His method of procedure may be judged of by the narration of a case which recently occurred at the Lariboisiére Hospital and was observed by all who attend there. A healthy man, aged nineteen, was admitted February 17th, presenting all the symptoms of an acute abscess of the axilla, which had been about a week in forming. On the 19th, chloroform having been given, a considerable quantity of well conditioned pus was discharged by the bistoury, pressure being exercised in all directions for the purpose of securing complete evacuation. The cavity of the abscess was next thoroughly washed out with water introduced through the tube of an irrigator, in order to bring away any remaining pus, the injection being continued until the water returned completely limpid. Pressure was again employed to force out every drop of the water and the orifice was strapped up. A large pad of charpie was introduced into the axilla in order to make pressure over that region, and the arm was confined in one of Mayor's bandages, as if for fracture of the clavicle. On the 21st cicatrization was complete, no discharge of pus whatever being visible. The bandage was continued as a matter of precaution for two or three days, and then the arm was allowed to hang down, no pain being reproduced. In the site of the abscess a little indurated spot could be felt. On the 27th he was discharged quite well.—*Gazette des Hôpitaux*, 1855.—*Ibid.*

